# Prevalence and Risk Factors of Iron Deficiency Anemia in Pregnancy in Ghana

**DOI:** 10.1002/fsn3.71293

**Published:** 2025-11-25

**Authors:** Betty Osei‐Ntiamoah, Yvonne Nartey, Kwadwo Ameyaw Korsah

**Affiliations:** ^1^ Department of Obstetrics and Gynaecology Ashanti Regional Hospital, Ghana Health Service Kumasi Ghana; ^2^ Department of Adult Health, School of Nursing and Midwifery University of Ghana Legon Ghana

**Keywords:** biopsychosocial model, ghana, iron deficiency anemia, pregnancy, prevalence, risk factors

## Abstract

Iron deficiency anemia (IDA) is highly prevalent in Ghana. Yet, knowledge of context‐specific risk factors remains limited. This study investigated the prevalence and risk factors of IDA among 394 pregnant women (aged 15–49 years) attending antenatal care at Ejura Municipal Hospital. A cross‐sectional design guided by Engel's biopsychosocial model was employed. Data were collected using a structured questionnaire and IDA status was determined via hemoglobin (Hb) levels or a full blood count, following the WHO definition of anemia (Hb < 11.0 g/dL). Overall and subgroup prevalence were estimated, and risk factors were analyzed using logistic regression. Participants' ages ranged from 15 to 46 years, with a mean of 29 years. About 46.5% were 30 years or older, 67.0% resided in Hiawoanwu and Mpaebo, and 48.7% were Ewe or Sisala. The prevalence of IDA was 81.0%, indicating a high burden. Unemployed women were 3.55 times more likely to be anemic than farmers (adjusted odds ratio [AOR] = 3.55, 95% CI: 1.41–8.91, *p* = 0.01), and women not taking malaria prophylaxis were more likely to be anemic than those who did (AOR = 2.19, 95% CI: 1.19–4.04, *p* = 0.01), after adjusting for age, ethnicity, and residence. The study identified both established and context‐specific risk factors, highlighting the need to revise educational guidelines, implement skills training, and replicate research in other settings. The high prevalence of IDA underscores its significance as a critical public health issue.

## Graphical abstract

*Key findings*: The study investigated the prevalence and risk factors of iron deficiency anemia (IDA) in pregnancy. A prevalence of 81.0% was reported, based on full blood count or hemoglobin levels, indicating a high burden of IDA in the study setting. Identified risk factors included unemployment, age, diet and malaria prophylaxis. *Conclusion*: The study findings underscore the urgency of introducing guidelines capable of curbing this burden.

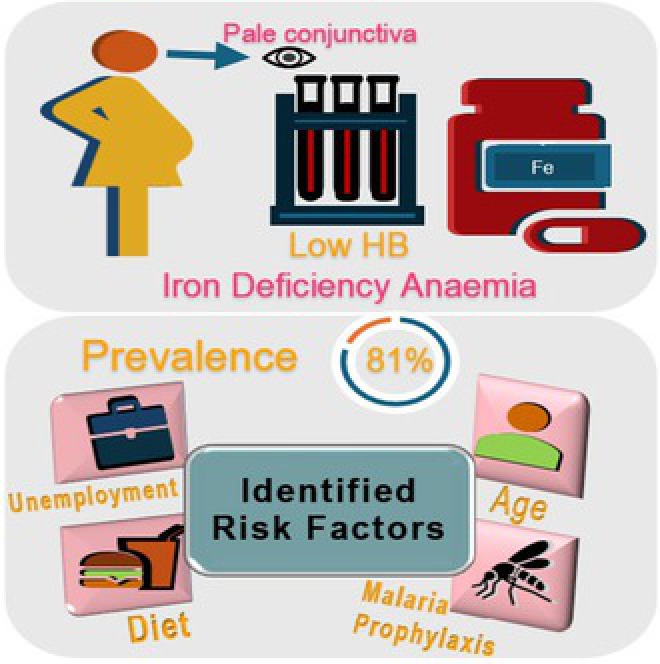

**Abbreviations**
ANC: antenatal care
AOR: adjusted odds ratio
CI: confidence interval
FBC: full blood count
g/dL: grams per deciliter
IDA: iron deficiency anemia
ITN: insecticide‐treated net
PPH: postpartum hemorrhage
UNFPA: United Nations Population Fund
UNICEF: United Nations International Children's Emergency Fund
WHO: World Health Organization

## 1 Introduction

Maternal mortality is a global health concern. It occurred both intrapartum and postpartum in approximately 287,000 women in 2020 (UNICEF 2021). Among these deaths, 95% were recorded in the least developed countries, with the majority being preventable. A mortality ratio of 12:100,000 live births was recorded in the high‐income countries, in contrast to 430:100,000 in the least developed countries (UNFPA 2020; WHO 2020). The leading causes are postpartum hemorrhage (PPH) (30%) and hypertensive disorders (14%) (Ghana Statistical Service (GSS), Ghana Health Service (GHS), and I 2018). PPH often results from altered blood coagulation due to severe anemia, causing a maternal death every 4 min globally, as reported by a study (Sebghati and Chandraharan 2017).

Anemia is a significant concern in pregnant women, defined as a hemoglobin concentration less than 11 g/dL (WHO 2014). It affects about 30%–37% of women and pregnant women aged 15–49 years. The majority of these cases are due to iron deficiency, increasing the possibility of miscarriage, premature birth, stillbirth, and light‐for‐date babies (WHO 2023). Iron deficiency anemia (IDA), characterized by insufficient red blood cells is common during pregnancy. The greater severity of IDA causes excessive bleeding and an increased tendency to complications (Faysal et al. 2023; Frass 2015; Omotayo et al. 2021; WHO 2024). In Africa and Asia, IDA and PPH are the major causes of death in 40%–43% of pregnant women (Lao et al. 2022). Iron deficiency forms 50% of anemia cases globally, particularly affecting females at the verge of reproduction in the least developed countries, contributing to high death rates of mothers and babies (Benson et al. 2021; Stevens et al. 2013).

Preventive measures include sleeping under an insecticide‐treated net (ITN), sulphadoxine‐pyrimethamine protocol adherence, anthelmintics and routine antenatal care supplements (USAID 2018). Hemoglobin or full blood count (FBC) measurements at the first antenatal appointment, ideally within the first 12 weeks, at 28 weeks and 36 weeks, are recommended for prompt diagnosis and treatment of IDA (Wemakor 2019). Despite these measures, the worldwide prevalence of IDA was 38.0% in 2020, with Africa accounting for 46.3%, compared to 18.0% in high‐income countries and between 35% and 75% in least developed countries (WHO 2021). Again, 35.6% was reported in a systematic review involving 15,061 pregnant women in sub‐Saharan Africa (Fite et al. 2021). Specifically in Ghana, IDA affected 51% of pregnant women in 2022 (GSS, GHS, and I 2023).

Several risk factors are associated with IDA in pregnancy, including inadequate iron intake, increased demand (such as in multigravida women), altered iron metabolism, low socioeconomic status, illiteracy, early marriage and higher susceptibility to infections such as hookworm (WHO 2021). While these are established risk factors, their impact in specific settings is less well understood. Therefore, studies in least developed countries are necessary to better understand these factors and develop targeted prevention strategies. Effective control of IDA in pregnant women in Ghana involves a detailed investigation into prevalence, associated risk factors, cultural influences, and health system responsiveness. The aim of this study was therefore to investigate the prevalence and risk factors of IDA in pregnant women in the Ejura Municipal Hospital, Ashanti Region of Ghana.

## 2 Materials and Methods

### 2.1 Aim, Study Design, Study Area, and Participants

The study aimed to determine the prevalence and risk factors of IDA in pregnant women at Ejura Municipal Hospital. A cross‐sectional study was conducted from January to December 2023, focusing on pregnant women aged 15–49 years receiving care at the study site. The Ashanti region, where Ejura is located, has an estimated population of 5,440,463 and is divided into 27 districts (Ghanaregions.com 2022). Ejura has a population of 137,672, including 69,121 women, according to the 2021 census. The Ejura Municipal Hospital functions as a referral center for surrounding towns such as Kasei, Atebubu, Yeji, and Nkoranza. The antenatal care (ANC) clinic is managed by 10 midwives providing services to approximately 120 clients daily. All the clinic registrants who consented to participate were recruited for the study.

### 2.2 Inclusion and Exclusion Factors

The study included women aged 15–49 years receiving ANC at Ejura Municipal Hospital, and fluent in either Twi or English. However, it excluded pregnant women seen at the general outpatient department or those who were ill.

### 2.3 Sample Size and Sampling Technique

The sample size was determined by the Charan and Biswas' formula for cross‐sectional studies (Charan and Biswas 2013). The outcome of a previous study at Kintampo Municipal Hospital was employed, and with a 63% IDA prevalence, a margin of error of 5% and a confidence level of 95% (*Z* = 1.96), a sample size of 358 was determined. 10% was added to arrive at 394 to cater for missing responses. ANC days were identified, excluding postnatal days, and participants were selected by convenience sampling on ANC days.

### 2.4 Data Collection

Data were collected with a semi‐structured questionnaire, guided by the constructs of Engel's biopsychosocial model, capable of probing into the condition rather than concentrating on only symptoms (Engel 1977). The questionnaire included sections on socio‐demographic, biological, psychological, social and environmental characteristics (See Document S1). Hemoglobin or FBC data, checked and recorded on the same day as questionnaire administration, were used to determine IDA status due to the unavailability of serum ferritin. Participants were given detailed information about the study objectives. Questionnaires were administered face‐to‐face by the main author and trained research assistants.

### 2.5 Quality Assurance

The questionnaire was initially designed in English, changed to Twi and then reverted to its original state for accuracy. It was pre‐tested on a similar population outside the study site. Research assistants received 2 days' training on data collection procedures, ethical considerations and participant rights, including voluntary participation and confidentiality. They were assessed for readiness before fieldwork began. Continuous monitoring was done to ensure consistency in data collection. Data collection was carried out by the main author and two trained health workers. Participants were given time to complete the questionnaire conveniently. All responses were reviewed for completeness before the participants left. Missing or unclear responses were clarified and corrected with the respondents' input.

### 2.6 Ethics Statement

Ethical approval was obtained from the Ghana Health Service Ethical Review Committee (GHS‐ERC: 058/09/32). Institutional permission was secured from hospital management. Participant information sheets were provided and explained, with consent obtained before data collection. For minors, parental or guardian consent was obtained. Participants were assured of confidentiality and informed that data would be used solely for this research. The study adhered to the Declaration of Helsinki (World Medical Association 1974).

### 2.7 Data Entry and Analysis

Data from the Excel file was transferred to STATA version 17.0 and cleaned for analysis. The study used descriptive statistics (frequencies and percentages) for demographic and clinical variables. Univariate logistic regression explored the association between the independent variables and IDA. A multivariate logistic regression analysis was also done, adjusting for age, ethnicity, and residence. A *p* < 0.05 was considered statistically significant. Data from 394 participants was analyzed with no missing variables.

## 3 Results

This section highlights the sociodemographic characteristics, overall prevalence and the prevalence based on the sociodemographic characteristics. It also explores the association between the various characteristics and IDA.

### 3.1 Socio‐Demographic Characteristics of Study Participants

The study included 394 pregnant women, with their sociodemographic factors displayed in Table 1. The majority of the women were aged 15–46 years, with a mean age of 29 years (SD ±7). It can be inferred that 11.4% of the women were below 20 years, 42.1% were aged 20–29 years, and 46.5% were aged 30 years and above. More than half (67.0%) resided in other areas of the municipality, and 8.1% resided in Sabonline. About 34.5% of the women were Akan, 9.4% were Dagomba, 7.4% were Gonja, 48.7% belonged to other ethnic groups, and 51.0% belonged to families of five or fewer. Regarding education, 26.6% had no formal education, 33.0% had completed Junior High School, 19.8% had completed primary education, 13.2% were Senior High School leavers, and 7.4% attended tertiary. In terms of occupation, a little above one‐third (33.5%) were unemployed, and 7.4% were formally employed.

**TABLE 1:** Socio‐demographic characteristics of the study participants.

Characteristics	*N* (394)	%
Age (group in years)		
Less than 20	45	11.4
20–29	166	42.1
30 or more	183	46.5
Residence		
Sabonline	32	8.1
Mempeasem/Dagombaline	40	10.2
Seko/Ejurafie/Badukrom	58	14.7
Others	264	67.0
Ethnicity		
Akan	136	34.5
Dagomba	37	9.4
Gonja	29	7.4
Others	192	48.7
Family size		
≤ 5	201	51.0
≥ 6	193	49.0
Educational level		
Primary	78	19.8
Junior high	130	33.0
Senior high	52	13.2
Tertiary	29	7.4
No formal education	105	26.6
Occupation		
Farmer	105	26.6
Unemployed	132	33.5
Formally employed	29	7.4
Informally employed	128	32.5
Marital status		
Married	258	65.5
Single	65	16.5
Divorced or cohabitation	71	18.0
Type of marriage		
Monogamy	210	53.3
Polygamy	48	12.2
Not applicable	136	34.5
Religious affiliation		
Christian	271	68.8
Muslim	120	30.4
Traditionalist	3	0.8
Others	0	0.0

### 3.2 Prevalence of IDA

The overall IDA rate in the antenatal clinic of Ejura Municipal Hospital was reported as 81.0%.

### 3.3 Prevalence of IDA According to Socio‐Demographic Characteristics

Among pregnant women aged ≥ 30 years (*n* = 183), anemia was observed in 149 cases (81.4%). Similarly, 127 out of 166 pregnant women aged 20–29 years were anemic, representing 76.5%. The highest prevalence was observed among women below 20 years, with 42 out of 45 affected (93.3%) (Table 2). Furthermore, pregnant women who lived in Mempeasem and Dagomba line had a higher anemia prevalence (90.0%), followed by Sabonline (87.5%), Seko, Ejurafie, and Badukrom (81.0%), and other areas of the Ejura Municipality (78.4%). About 77.2% of the Akan, 81.1% of the Dagomba, 86.2% of the Gonja, and 82.3% of the other ethnic groups were anemic. A total of 70 (89.7%) pregnant women who completed primary school, 84.6% of those who attended Junior High School, 67.3% of those who completed Senior High School, 51.7% of those who completed tertiary education, and 83.8% of those with no formal education were anemic. Anemia prevalence was higher among the unemployed (92.4%), followed by farmers (81.0%), informally employed (76.6%), and formally employed (44.8%).

**TABLE 2:** Prevalence of IDA according to the socio‐demographic characteristics.

Characteristics	Anemic	Population	Prevalence (%)
Age			
< 20	42	45	93.3
20–29	127	166	76.5
≥ 30	149	183	81.4
Residence			
Sabonline	28	32	87.5
Mempeasem/Dagomba line	36	40	90.0
Seko/Ejurafie/Badukrom	47	58	81.0
Others	207	264	78.4
Ethnicity			
Akan	105	136	77.2
Dagomba	30	37	81.1
Gonja	25	29	86.2
Others	158	192	82.3
Family size			
≤ 5	150	201	74.6
≥ 6	168	193	87.0
Educational level			
Primary	70	78	89.7
Junior high	110	130	84.6
Senior high	35	52	67.3
Tertiary	15	29	51.7
No formal education	88	105	83.8
Occupation			
Farmer	85	105	81.0
Unemployed	122	132	92.4
Formally employed	13	29	44.8
Informally employed	98	128	76.6
Marital status			
Married	197	258	76.4
Single	57	65	87.7
Divorce or cohabitation	64	71	90.0
Type of marriage			
Monogamy	153	210	72.9
Polygamy	44	48	91.7
Not applicable	121	136	89.0
Religious affiliation			
Christian	213	271	78.6
Muslim	102	120	85.0
Traditionalist	3	3	100.0

### 3.4 Risk Factors of IDA

The study further examined risk factors for IDA based on the sociodemographic data of pregnant women.

#### 3.4.1 Association Between Sociodemographic Characteristics and IDA

Table 3 summarizes the association between sociodemographic characteristics and IDA. It was revealed that pregnant women aged 25–29 years were 0.47 times less likely to have IDA than those below 25 years, after adjusting for ethnicity and residence (adjusted odds ratio [AOR] = 0.47; 95% CI: 0.23–0.97). Unemployed women were 3.55 times more likely to have IDA (AOR = 3.55; 95% CI: 1.41–8.91), formally employed women were 0.19 times less likely to have IDA (AOR = 0.19; 95% CI: 0.07–0.49) than farmers. Pregnant women who had completed Senior High School were 0.29 times less likely to be anemic (AOR = 0.29; 95% CI: 0.11–0.79) than those completing primary education, after adjusting for the factors mentioned above.

**TABLE 3:** Association between socio‐demographic characteristics and iron deficiency anaemia.

Characteristics	Not anemic (*N* = 76)	Anemic (*N* = 318)	Unadjusted OR (95% CI)	Adjusted OR (95% CI)
Age (group in years)				
< 25	13 (17.1)	85 (26.7)	1.00	1.00
25–29	29 (38.2)	84 (26.4)	0.44 (0.22–0.91)	0.47 (0.23–0.97)
30 or more	34 (44.7)	149 (46.9)	0.67 (0.34–1.34)	0.69 (0.34–1.38)
Residence				
Sabonline/Mempeasem/Dagombaline	8 (10.5)	64 (20.1)	1.00	1.00
Seko/Ejurafie/Badukrom	11 (14.5)	47 (14.8)	0.53 (0.20–1.43)	0.63 (0.22–1.79)
Others	57 (75.0)	207 (65.1)	0.45 (0.21–1.00)	0.50 (0.22–1.12)
Ethnicity				
Akan	31 (40.8)	105 (33.0)	1.00	1.00
Dagomba/Gonja	11 (14.5)	55 (17.3)	1.48 (0.69–3.16)	1.32 (0.59–2.97)
Others	34 (44.7)	158 (49.7)	1.37 (0.79–2.37)	1.29 (0.74–2.29)
Occupation				
Farmer	20 (26.3)	85 (26.7)	1.00	1.00
Unemployed	10 (13.2)	122 (38.4)	3.97 (1.28–6.44)	3.55 (1.41–8.91)
Formally employed	16 (21.0)	13 (4.1)	0.38 (0.08–0.46)	0.19 (0.07–0.49)
Informally employed	30 (39.5)	98 (30.8)	0.95 (0.41–1.45)	0.74 (0.38–1.45)
Family size?				
≤ 5	51 (67.1)	150 (47.2)	1.00	1.00
≥ 6	25 (32.9)	168 (52.8)	2.28 (1.35–3.87)	1.81 (0.99–3.30)
Educational level				
Primary	8 (10.5)	70 (22.0)	1.00	1.00
Junior high	20 (26.3)	110 (34.6)	0.63 (0.26–1.50)	0.61 (0.25–1.53)
Senior high	17 (22.4)	35 (11.0)	0.24 (0.09–0.60)	0.29 (0.11–0.79)
Tertiary	14 (18.4)	15 (4.7)	0.12 (0.04–0.34)	0.36 (0.07–1.88)
No formal education	17 (22.4)	88 (27.7)	0.59 (0.24–1.45)	0.61 (0.23–1.60)
Marital status				
Married	61 (80.3)	197 (62.0)	1.00	1.00
Single	8 (10.5)	57 (17.9)	2.21 (1.00–4.88)	1.04 (0.36–2.99)
Divorced and cohabitation	7 (9.2)	64 (20.1)	2.83 (1.23–6.50)	2.68 (1.02–7.02)

*Note:* Adjusted for age, residence and ethnicity.

#### 3.4.2 Association Between Biological Characteristics and IDA

Table 4 shows the association between the biological characteristics and IDA. It can be seen that those who had been on hospital admission were 5.35 times more likely to have IDA than not being hospitalized in the current pregnancy, after adjusting for age, ethnicity and residence (AOR = 5.35; 95% CI: 1.26–22.82). Pregnant women who had not taken malaria prophylaxis were 2.19 times more likely to have IDA than those who received it (AOR = 2.19; 95% CI: 1.19–4.04). Those who were not using ITN were 0.47 times less likely to have IDA than those using ITN (AOR = 0.47; 95% CI: 0.23–0.96). Those who were not attending ANC regularly were 2.51 times more likely to have IDA than regular attendants (AOR = 2.51; 95% CI: 1.01–6.26).

**TABLE 4:** Association between biological characteristics and iron deficiency anaemia.

Characteristics	Not anemic (*N* = 76)	Anemic (*N* = 318)	Unadjusted OR (95% CI)	Adjusted OR (95% CI)
Malaria prophylaxis				
Yes	60 (78.9)	192 (60.4)	1.00	1.00
No	16 (21.1)	126 (39.6)	2.46 (1.36–4.46)	2.19 (1.19–4.04)
Number of meals per day				
≥ 3	68 (89.5)	195 (61.3)	1.00	1.00
≤ 2	8 (10.5)	123 (38.7)	5.36 (2.49–11.54)	4.17 (1.88–9.26)
Vegetables taken per week				
≥ 3	32 (42.1)	67 (21.1)	1.00	1.00
≤ 2	44 (57.9)	246 (77.4)	2.81 (1.69–4.70)	2.14 (1.20–3.80)
Meat taken per week				
≥ 3	59 (77.6)	164 (51.6)	1.00	1.00
≤ 2	17 (22.4)	154 (48.4)	3.26 (1.82–5.84)	1.82 (0.90–3.70)
Milk taken per week				
≥ 3	32 (42.1)	37 (11.6)	1.00	1.00
≤ 2	44 (57.9)	281 (88.4)	5.52 (3.12–9.77)	2.93 (1.46–5.88)
Eggs taken per week				
≥ 3	50 (65.8)	149 (46.9)	1.00	1.00
≤ 2	26 (34.2)	169 (53.1)	2.18 (1.29–3.68)	1.02 (0.54–1.94)
Tea consumption				
No	41 (53.9)	182 (57.2)	1.00	1.00
Yes	35 (46.1)	136 (42.8)	0.88 (0.53–1.45)	1.70 (0.87–3.31)
Contraceptive use				
Yes	31 (40.8)	108 (34.0)	1.00	**1.00**
No	45 (59.2)	210 (66.0)	1.34 (0.80–2.24)	2.12 (1.00–4.49)
Abnormal vagina discharge				
No	65 (85.5)	201 (63.2)	1.00	1.00
Yes	11 (14.5)	117 (36.8)	3.44 (1.75–6.78)	3.19 (1.47–6.89)
MUAC				
16–25.74 (underweight)	15 (19.7)	95 (29.9)	1.00	1.00
25.75–28.10 (normal)	22 (28.9)	122 (38.4)	0.88 (0.43–1.78)	1.41 (0.60–3.32)
28.11–30.25 (overweight)	18 (23.7)	50 (15.7)	0.44 (0.20–0.94)	0.76 (0.30–1.91)
30.26–38 (obese)	21 (27.6)	51 (16.0)	0.38 (0.18–0.81)	0.65 (0.24–1.71)
BMI				
Normal	30 (39.5)	154 (48.4)	1.00	1.00
Underweight/overweight/obese	46 (60.5)	164 (51.6)	0.69 (0.42–1.16)	1.13 (0.50–2.58)
Gestational age				
≤ 27	28 (36.8)	133 (41.8)	1.00	1.00
28–42	48 (63.2)	185 (58.2)	0.81 (0.48–1.36)	0.74 (0.32–1.72)
Gestational age at the first visit				
≤ 12	37 (48.7)	104 (32.7)	1.00	1.00
13–41	39 (51.3)	214 (67.3)	1.95 (1.18–3.24)	0.93 (0.43–2.00)
Number of ANC visits				
≥ 5	28 (36.8)	95 (29.9)	1.00	1.00
1–2	20 (26.3)	129 (40.6)	1.90 (1.01–3.58)	0.31 (0.07–1.46)
3–4	28 (36.8)	94 (29.6)	0.99 (0.54–1.80)	0.58 (0.22–1.49)

*Note:* Adjusted for age, residence and ethnicity.

Abbreviations: ANC, antenatal clinic; BMI, body mass index; ITN, insecticide‐treated net; MUAC, mid‐upper arm circumference.

Furthermore, pregnant women who consumed two or fewer meals daily were 4.17 times more likely to have IDA than those consuming ≥ 3 meals (AOR = 4.17; 95% CI: 1.88–9.26). Those who consumed vegetables two times or fewer per week were 2.14 times more likely to have IDA than those consuming vegetables three times or more (AOR = 2.14; 95% CI: 1.20–3.80). Those who consumed milk two times or fewer per week were 2.93 times more likely to have IDA than those consuming milk three times or more (AOR = 2.93; 95% CI: 1.46–5.88). Those who had abnormal vaginal discharge were 3.19 times more likely to have IDA than those without it (AOR = 3.19; 95% CI: 1.47–6.89), after adjusting for the factors mentioned above.

#### 3.4.3 Association Between Psychological Characteristics and IDA

Pregnant women whose beliefs discouraged them from eating certain nutritious foods were 1.27 times more likely to develop IDA compared to those without such beliefs (AOR = 1.27; 95% CI: 0.55–2.93) (Table 5). Those who experienced mood swings were 1.42 times more likely to have IDA than those who had no such experience (AOR = 1.42; 95% CI: 0.77–2.64). Those whose religion restricted them from eating certain foods were 1.02 times more likely to have IDA than those whose religion had no such restrictions (AOR = 1.02; 95% CI: 0.48–2.14). None of the psychological characteristics were significant in both the unadjusted and adjusted models.

**TABLE 5:** Association between psychological/social/environmental characteristics and iron deficiency anemia.

Characteristics	Not anemic (*N* = 76)	Anemic (*N* = 318)	Unadjusted OR (95% CI)	Adjusted OR (95% CI)
Belief restrictions				
No	68 (89.5)	270 (84.9)	1.00	1.00
Yes	8 (10.5)	48 (15.1)	1.51 (0.68–3.34)	1.27 (0.55–2.93)
Experience mood swings				
No	60 (78.9)	232 (73.0)	1.00	1.00
Yes	16 (21.1)	86 (27.0)	1.39 (0.76–2.54)	1.42 (0.77–2.64)
Religion restrictions				
No	61 (80.3)	238 (74.8)	1.00	1.00
Yes	15 (19.7)	80 (25.2)	1.37 (0.74–2.54)	1.02 (0.48–2.14)
Role restrictions				
No	71 (93.4)	223 (70.1)	1.00	1.00
Yes	5 (6.6)	95 (29.9)	6.05 (2.37–15.46)	7.35 (2.77–19.51)
Income per month				
901–5000	18 (23.7)	14 (4.4)	1.00	1.00
≤ 900	25 (32.9)	127 (39.9)	6.74 (3.17–14.31)	6.02 (2.58–14.01)
Economic effect				
No	57 (75.0)	145 (45.6)	1.00	1.00
Yes	19 (25.0)	173 (54.4)	3.58 (2.04–6.29)	3.51 (1.97–6.26)
Media effect				
No	64 (84.2)	217 (68.2)	1.00	1.00
Yes	12 (15.8)	101 (31.8)	2.48 (1.28–4.80)	1.75 (0.86–3.55)

*Note:* Adjusted for age, residence and ethnicity.

#### 3.4.4 Association Between Social Characteristics and IDA

The study reported that pregnant women whose roles restricted their ability to eat well were 7.35 times more likely to have IDA than women whose roles had no such restrictions (AOR = 7.35; 95% CI: 2.77–19.51) (Table 5). Also, earning 900 cedis or less was 6.02 times more likely to have IDA than earning from 901 to 5000 cedis (AOR = 6.02; 95% CI: 2.58–14.01), after adjusting for age, ethnicity, and residence.

#### 3.4.5 Association Between Environmental Characteristics and IDA

Finally, pregnant women who indicated that the economy affected their ability to patronize foods they had received education on were 3.51 times more likely to have IDA than those without such concerns (AOR = 3.51; 95% CI: 1.97–6.26), as indicated in Table 5.

## 4 Discussion

The current study investigated the prevalence and risk factors of IDA in pregnancy at Ejura Municipal Hospital. The prevalence was 81.0%, with variations observed across sociodemographic and clinical factors such as age, occupation, education, hospital admission, malaria prophylaxis, ANC attendance, milk intake, meal frequency, income, abnormal vaginal discharge, role restrictions and economic conditions. The unusually high prevalence may partly be explained by the heavy patronage of the hospital's laboratory, as other facilities in the district lack well‐equipped laboratories. Many pregnant women referred for laboratory services subsequently registered at the hospital's ANC. Additionally, the measurement method may have influenced results, since low hemoglobin levels do not always reflect iron deficiency.

IDA prevalence among pregnant women in Ejura Municipal Hospital was higher than the 33% reported in the Volta Region (Kofie et al. 2019) and 62.6% in the Northern Region of Ghana (Agyeman et al. 2021). Globally, about 38% of pregnant women are anemic, with Africa accounting for 46.3% of this total. High‐income countries recorded a prevalence of 18% while least developed countries recorded 35%–75%, which remains lower than the prevalence reported in this study (WHO 2021). Additionally, IDA prevalence in Ghana is generally lower than in certain regions or communities, which contrasts with the high rate in Ejura. This could be attributed to demographic factors, such as a large number of less educated pregnant women. The area is also home to various ethnic groups from other parts of the country who may not be fluent in Twi or English, which can hinder comprehension of health education.

The findings in this study are higher than those of a cross‐sectional study in Nigeria (41.0%) (Babah 2024), 26.4% in Ethiopia (Geta et al. 2022), 29.0%–42.7% in South Africa (Turawa et al. 2021), and 18.0% in Tanzania (Stephen et al. 2018). Generally, IDA prevalence varies across study areas due to geographic and cultural factors. Nevertheless, the high burden observed in this setting warrants attention due to its impact on maternal health, as well as complications such as preterm birth and intrauterine growth restriction (Daru et al. 2018). Further investigation is needed to better understand the underlying causes and contextual drivers of this elevated prevalence.

Women aged 30 years or older had the highest prevalence (46.9%), followed by those under 25 years (26.7%) and those aged 25–29 years (26.4%). The differences across age groups may be due to the number of respondents in each category and the influence of other factors. The younger age groups had the smallest sample sizes (84 and 85, respectively), which may explain the lower prevalence. Another reason might be that most of the younger women were first‐time mothers who reported to the clinic early out of curiosity or anxiety, unlike older women, many of whom had been pregnant or delivered before. Regular ANC attendance enables access to ITN, malaria prophylaxis, iron supplements, and nutrition education, which help prevent IDA. This may account for the lower prevalence in younger women. These findings align with studies in Tanzania and a comparative study between Indonesia and Ghana, which revealed similar outcomes (Ali et al. 2019; Mocking et al. 2018).

Akowuah et al. attributed higher IDA prevalence among women aged ≥ 30 years to poor feeding habits and prior safe deliveries, which may lead them to ANC delay (Akowuah et al. 2022). In contrast to this study, others found higher IDA prevalence among teenagers (Annan et al. 2021; Obeagu and Agreen 2023). Age was significantly associated with IDA, consistent with a study in China, though the significance there was in women aged 35 and above (Lin et al. 2018). However, a study in Indonesia recorded no significant link between age and IDA (Lestari et al. 2018).

Unemployed pregnant women recorded the highest IDA prevalence (38.4%), followed by those in informal employment (30.8%), farmers (26.7%), and formally employed women (4.1%). Differences in category populations may partly explain the low prevalence among the formally employed. This may also reflect the generally low literacy rate in Ejura, with few formally employed individuals who are more likely to understand ANC education. This suggests that if more women were formally employed, the prevalence of IDA in the setting would have been lower because they could better understand information. Unemployment led to a 3.55‐fold higher odds of IDA over farmers, possibly due to income constraints preventing them from buying nutrient‐rich foods. A significant association was also found between formal employment and lower IDA. Although farming was not linked to IDA significantly, a study in Indonesia reported such a connection, possibly due to poor nutritional knowledge (Lestari et al. 2018). Employment generally provides income, increasing the likelihood of affording essential supplements when shortages occur in hospitals. Without employment or external support, affording these essentials becomes difficult.

A family size of greater than or equal to six led to an increased IDA rate (52.8%) compared to families with five or fewer members (47.2%). Although not statistically significant, larger families increase the chance of IDA by 1.81 times. This result is consistent with research in Saudi Arabia and Bangladesh, which linked larger families with increased IDA risk (Ahmed et al. 2019; Alreshidi and Haridi 2021). Larger families may experience food insecurity, making it harder to meet the elevated iron demands in their gravid state. However, some studies did not find a link between family size and IDA (Akowuah et al. 2022; Sabina Azhar et al. 2021), possibly due to geographical or socio‐economic differences.

IDA prevalence was highest among women with junior high education (34.6%), followed by those with no formal education (27.7%), primary education (22.0%), senior high school (11.0%), and tertiary education (4.7%). A significant association was found between senior high school completion and lower IDA risk. Education likely enhances the ability to understand nutritional advice given during ANC. These findings are supported by a study showing that secondary or higher education lowers IDA risk (Stephen et al. 2018), though a study in China found no link between secondary education and IDA (Lin et al. 2018).

Hospital admission increased the odds of having IDA by 5.35 times. Admissions can stem from various conditions, and post‐discharge complications may increase IDA risk. For example, a woman admitted for hypertension might contract an unnoticed infection that contributes to IDA. Healthy lifestyles and compliance with prescribed treatments are essential to prevent complications and hospitalizations. Not taking malaria prophylaxis was significantly associated with IDA, consistent with findings from Akatsi South in Ghana (Ahadzie‐Soglie et al. 2022). However, another study in Ghana found no significant association (Agyeman et al. 2021). Pregnant women must be encouraged to take malaria prophylaxis regularly to prevent malaria and its complications, including IDA.

Non‐attendance at ANC was also significantly associated with IDA, consistent with findings by Saaka et al., who observed lower IDA risk among at least four times ANC attendants (Saaka et al. 2017). ANC offers key services like supplements, diagnostics, and education. Missing these services increases the risk of IDA. Women who ate two or fewer meals daily were 4.17 times more likely to have IDA. Proper nutrition is critical during pregnancy, which demands at least three meals daily, with one extra meal recommended. Factors like low income, morning sickness, or pica may reduce meal frequency. This supports findings from Bangladesh (Ahmed et al. 2019).

Milk intake was generally low, and consuming milk two or fewer times per week increased IDA risk by 2.93 times. Possible reasons include lack of awareness, lactose intolerance, or cultural taboos. Similar findings were reported in a study conducted in Pakistan (Hameed et al. 2018). Abnormal vaginal discharge also raised the odds of IDA by 3.19 times. This may be due to poor perineal hygiene or changes in genital mucosa during pregnancy. Infections can reduce iron absorption, hindering red blood cell production. A study conducted in Saudi Arabia reported a similar outcome (Khadawardi 2020).

Few women reported role restrictions, indicating many received household support. However, women whose roles restricted their ability to eat well were 7.35 times more likely to have IDA. Role overload may lead to food insecurity and depleted iron stores. This finding aligns with another Ghanaian study (Annan et al. 2021). Minimizing chores in pregnant women is important for their nutrition and health. Few participants were formally employed. Formal employment generally provides income for buying nutrient‐rich foods. Women earning GHS 900 or less were 6.02 times more likely to have IDA than those earning GHS 901–5000. This supports findings from Bangladesh and China (Lin et al. 2018; Sabina Azhar et al. 2021), though no significant link was found in another study (Getahun et al. 2017).

Pregnant women who said economic conditions hindered their ability to purchase recommended foods were 3.51 times more likely to have IDA, a statistically significant finding. This aligns with Lin et al. (Lin et al. 2018). Economic hardship affects employment, food access, and healthcare utilization. Strengthening the economy may improve dietary access and reduce IDA risk. In contrast, none of the psychological factors were statistically significant in this study. Although these variables are established contributors to IDA, most of the women reported not experiencing them. Possible explanations include limitations of the measurement tool, participants' limited knowledge of these factors, or contextual influences related to the study setting.

Although the questionnaire did not include a specific section on barriers to maintaining appropriate hemoglobin levels, factors such as non‐attendance (possibly due to distance), diet, family size, vaginal infections, and non‐use of sulphadoxine‐pyrimethamine were considered relevant. Some of these factors are consistent with findings from a study in the Adaklu District of Ghana (Tettegah et al. 2023). It is important to emphasize that the findings discussed represent associations rather than causal relationships.

## 5 Strengths and Limitations

The relatively high number of participants who agreed to take part was a strength of the study, as was its status as the first of its kind in this setting, providing comprehensive insights into IDA risk factors and prevalence. These findings can guide local strategies for addressing IDA. However, the study had limitations. It focused on one facility due to resource constraints, excluding women from other health centers in the municipality. Some educated participants may have sought online help when answering the questionnaire, possibly skewing the results. It also excluded pregnant women attending out‐patient department directly rather than ANC.

The study considered meal frequency but not meal content or portion size. It also did not assess malaria status or supplement adherence. Hemoglobin levels and FBC were used as proxies for determining the IDA status, as serum ferritin testing was unavailable at the facility. This may have introduced bias, since not all cases of low hemoglobin are attributable to IDA. Nonetheless, evidence suggests that the majority of anemia cases in pregnancy are due to IDA. Laboratory data relied on records in ANC books, which may contain errors. The small sample size limited the analysis of certain risk factors and anemia severity. Nonetheless, the study revealed a high IDA prevalence and valuable insights for policymaking.

## 6 Conclusions

The prevalence of IDA among pregnant women in the Ejura Municipal Hospital was high, underscoring its significant burden in this population. Contributing factors included age, occupation, education, hospital admission, malaria prophylaxis, ANC attendance, milk intake, meal frequency, income, abnormal vaginal discharge, role restrictions, and economic hardship. Addressing this public health concern will require multifaceted strategies. Encouraging pregnant women to engage in income‐generating activities may help them manage healthcare costs during periods of shortage, while vocational training offers another viable pathway. Government action to reduce hospital supply shortages is also essential. Future research should test interventions targeting these risk factors and replicate this study in other districts or regions to identify context‐specific needs and inform policy.

## Author Contributions

**Betty Osei‐Ntiamoah:** conceptualization (lead), data curation (lead), formal analysis (lead), investigation (lead), methodology (lead), project administration (lead), validation (lead), visualization (lead), writing – original draft (lead), writing – review and editing (equal). **Yvonne Nartey:** conceptualization (equal), data curation (equal), formal analysis (equal), methodology (equal), project administration (equal), supervision (equal), validation (equal), visualization (equal), writing – review and editing (equal). **Kwadwo Ameyaw Korsah:** conceptualization (equal), data curation (equal), formal analysis (equal), methodology (equal), project administration (equal), supervision (equal), validation (equal), visualization (equal), writing – review and editing (equal).

## Funding

The authors have nothing to report.

## Ethics Statement

The study received ethics approval from the Ghana Health Service Ethical Review Committee (GHS‐ERC: 058/09/32). An introductory letter from the main author's institution was sent to the study area for approval before initiating data collection.

## Consent

Written informed consent was obtained from the pregnant women before administering the questionnaires. Informed consent was also sought from the parents or legal guardians of pregnant women who were minors before involving them in the study. Participants were assured of confidentiality and that the information they provided would be used solely for the study. The study complied with the provisions of the Declaration of Helsinki.

## Conflicts of Interest

The authors declare no conflicts of interest.

## Supporting information


**Data S1:** Supporting Information.

## Data Availability

The datasets used and/or analyzed during the current study are available from the corresponding author on request.

## References

[fsn371293-bib-0001] Agyeman, Y. N. , S. Newton , R. B. Annor , and E. Owusu‐Dabo . 2021. “Intermittent Preventive Treatment Comparing Two Versus Three Doses of Sulphadoxine Pyrimethamine (IPTp‐SP) in the Prevention of Anaemia in Pregnancy in Ghana: A Cross‐Sectional Study.” PLoS One 16, no. 4 April: 1–17. 10.1371/journal.pone.0250350. PMC8057609 33878140

[fsn371293-bib-0002] Ahadzie‐Soglie, A. , O. Addai‐Mensah , A. Abaka‐Yawson , A. M. Setroame , and P. K. Kwadzokpui . 2022. “Prevalence and Risk Factors of Malaria and Anaemia and the Impact of Preventive Methods Among Pregnant Women: A Case Study at the Akatsi South District in Ghana.” PLoS One 17, no. 7: 1–19. 10.1371/journal.pone.0271211. PMC9312417 35877761

[fsn371293-bib-0003] Ahmed, S. , M. A. Al Mamun , N. Mahmud , et al. 2019. “Prevalence and Associated Factors of Anaemia Among Pregnant Women Receiving Antenatal Care (ANC) at Fatima Hospital in Jashore, Bangladesh: A Cross‐Sectional Study.” Food and Nutrition Sciences 10, no. 9: 1056–1071. 10.4236/fns.2019.109076.

[fsn371293-bib-0004] Akowuah, J. A. , E. Owusu‐Addo , and A. A. Opuni . 2022. “Predictors of Anaemia Prevalence Among Ghanaian Pregnant Women: A Cross‐Sectional Study.” Inquiry: The Journal of Health Care Organization, Provision, and Financing 59: 1–8. 10.1177/00469580221086919. PMC9081711 35510934

[fsn371293-bib-0005] Ali, M. , A. F. Ngowi , and N. S. Gibore . 2019. “Prevalence and Obstetric Factors Associated With Anaemia Among Pregnant Women, Attending Antenatal Care in Unguja Island, Tanzania.” International Journal of Community Medicine and Public Health 6, no. 3: 950. 10.18203/2394-6040.ijcmph20190577.

[fsn371293-bib-0006] Alreshidi, M. A. , and H. K. Haridi . 2021. “Prevalence of Anaemia and Associated Risk Factors Among Pregnant Women in an Urban Community in the North of Saudi Arabia.” Journal of Preventive Medicine and Hygiene 62, no. 3: E653–E663. 10.15167/2421-4248/jpmh2021.62.3.1880. 34909493 PMC8639134

[fsn371293-bib-0007] Annan, R. A. , L. A. Gyimah , C. Apprey , et al. 2021. “Factors Associated With Iron Deficiency Anaemia Among Pregnant Teenagers in Ashanti Region, Ghana: A Hospital‐Based Prospective Cohort Study.” PLoS One 16, no. 4: 1–20. 10.1371/journal.pone.0250246. PMC8078754 33905433

[fsn371293-bib-0008] Babah, O. A. 2024. “Prevalence of and Risk Factors for Iron Deficiency Among Pregnant Women With Moderate or Severe Anaemia in Nigeria: A Cross‐Sectional Study.” BMC Pregnancy and Childbirth 24, no. 1: 39. 10.1186/s12884-023-06169-1. 38182997 PMC10768359

[fsn371293-bib-0009] Benson, C. S. , A. Shah , M. C. Frise , and C. J. Frise . 2021. “Iron Deficiency Anaemia in Pregnancy: A Contemporary Review.” Obstetric Medicine 14, no. 2: 67–76. 10.1177/1753495X20932426. 34394714 PMC8358243

[fsn371293-bib-0010] Charan, J. , and T. Biswas . 2013. “How to Calculate Sample Size for Different Study Designs in Medical Research?” Indian Journal of Psychological Medicine 35, no. 2: 121–126. 10.4103/0253-7176.116232. 24049221 PMC3775042

[fsn371293-bib-0011] Daru, J. , J. Zamora , B. M. Fernández‐Félix , et al. 2018. “Risk of Maternal Mortality in Women With Severe Anaemia During Pregnancy and Postpartum: A Multilevel Analysis.” Lancet Global Health 6, no. 5: e548–e554. 10.1016/S2214-109X(18)30078-0. 29571592

[fsn371293-bib-0012] Engel, G. L. 1977. “The Need for a New Medical Model: A Challenge for Biomedicine.” Science 196, no. 4286: 129–136. 847460 10.1126/science.847460

[fsn371293-bib-0013] Faysal, H. , T. Araji , and H. K. Ahmadzia . 2023. “Recognizing Who Is at Risk for Postpartum Haemorrhage: Targeting Anaemic Women and Scoring Systems for Clinical Use.” American Journal of Obstetrics and Gynecology MFM 5, no. 2: 100745. 10.1016/j.ajogmf.2022.100745. 36075528

[fsn371293-bib-0014] Fite, M. B. , N. Assefa , and B. Mengiste . 2021. “Prevalence and Determinants of Anemia Among Pregnant Women in Sub‐Saharan Africa: A Systematic Review and Meta‐Analysis.” Archives of Public Health 79, no. 1: 1–11. 10.1186/s13690-021-00711-3. 34861892 PMC8643002

[fsn371293-bib-0015] Frass, K. A. 2015. “Postpartum Haemorrhage Is Related to the Haemoglobin Levels at Labour: Observational Study.” Alexandria Journal of Medicine 51, no. 4: 333–337. 10.1016/j.ajme.2014.12.002.

[fsn371293-bib-0016] Geta, T. G. , S. Gebremedhin , and A. O. Omigbodun . 2022. “Prevalence and Predictors of Anemia Among Pregnant Women in Ethiopia: Systematic Review and Meta‐Analysis.” PLoS One 17, no. 7: 1–22. 10.1371/journal.pone.0267005. PMC9328503 35895619

[fsn371293-bib-0017] Getahun, W. , T. Belachew , and A. D. Wolide . 2017. “Burden and Associated Factors of Anaemia Among Pregnant Women Attending Antenatal Care in Southern Ethiopia: Cross Sectional Study.” BMC Research Notes 10, no. 1: 1–7. 10.1186/s13104-017-2605-x. 28705235 PMC5512984

[fsn371293-bib-0018] Ghana Statistical Service (GSS), Ghana Health Service (GHS), and I . 2018. “Ghana Maternal Health Survey 2017.” 10.1017/CBO9781107415324.004.

[fsn371293-bib-0020] Ghanaregions.com . 2022. “Ashanti Region Profile.” Accessed February 7, 2023. https://ghanaregions.com.

[fsn371293-bib-0019] GSS, GHS, and I . 2023. “Ghana Demographic and Health Survey 2022: Key Indicattors Report. Accra, Ghana, and Rockville, Maryland, USA: GSS and ICF., 5–24.”

[fsn371293-bib-0021] Hameed, H. , A. Hameed , S. Bashir , S. Akram , M. Arshad , and R. Afzal . 2018. “Study of Prevalence of Anaemia Among Pregnant Women and Its Correlation With Different Risk Factors.” Drug Designing: Open Access 7, no. 1: 1–5. 10.4172/2169-0138.1000158.

[fsn371293-bib-0022] Khadawardi, K. 2020. “Prevalence of Abnormal Vaginal Discharge Among Pregnant Women.” Medical Journal of Cairo University 88, no. 3: 677–683. 10.21608/mjcu.2020.104625.

[fsn371293-bib-0023] Kofie, P. , E. E. Tarkang , E. Manu , et al. 2019. “Prevalence and Associated Risk Factors of Anaemia Among Women Attending Antenatal and Post‐Natal Clinics at a Public Health Facility in Ghana.” BMC Nutrition 5, no. 1: 1–9. 10.1186/s40795-019-0303-x. 32153953 PMC7050900

[fsn371293-bib-0024] Lao, T. T. , L. L. Wong , S. Y. A. Hui , and D. S. Sahota . 2022. “Iron Deficiency Anaemia and Atonic Postpartum Haemorrhage Following Labour.” Reproductive Sciences 29, no. 4: 1102–1110. 10.1007/s43032-021-00534-1. 34993930

[fsn371293-bib-0025] Lestari, S. , I. I. Fujiati , D. Keumalasari , M. Daulay , S. J. Martina , and S. Syarifah . 2018. “The Prevalence of Anaemia in Pregnant Women and Its Associated Risk Factors in North Sumatera, Indonesia.” IOP Conference Series: Earth and Environmental Science 125, no. 1: 1–6. 10.1088/1755-1315/125/1/012195.

[fsn371293-bib-0026] Lin, L. , Y. Wei , W. Zhu , et al. 2018. “Prevalence, Risk Factors and Associated Adverse Pregnancy Outcomes of Anaemia in Chinese Pregnant Women: A Multicentre Retrospective Study.” BMC Pregnancy and Childbirth 18, no. 1: 1–8. 10.1186/s12884-018-1739-8. 29685119 PMC5914057

[fsn371293-bib-0027] Mocking, M. , A. I. Savitri , C. S. P. M. Uiterwaal , et al. 2018. “Does Body Mass Index Early in Pregnancy Influence the Risk of Maternal Anaemia? An Observational Study in Indonesian and Ghanaian Women.” BMC Public Health 18, no. 1: 1–9. 10.1186/s12889-018-5704-2. PMC6045841 30005609

[fsn371293-bib-0028] Obeagu, E. I. , and F. C. Agreen . 2023. “Anaemia Among Pregnant Women: A Review of African Pregnant Teenagers.” Public Health Nutrition 6, no. 1: 138. 10.35841/aajphn-6.1.138.

[fsn371293-bib-0029] Omotayo, M. O. , A. I. Abioye , M. Kuyebi , and A. C. Eke . 2021. “Prenatal Anaemia and Postpartum Haemorrhage Risk: A Systematic Review and Meta‐Analysis.” Journal of Obstetrics and Gynaecology Research 47, no. 8: 2565–2576. 10.1111/jog.14834. 34002432 PMC9258034

[fsn371293-bib-0030] Saaka, M. , J. Oladele , A. Larbi , and I. Hoeschle‐Zeledon . 2017. “Dietary Diversity Is Not Associated With Haematological Status of Pregnant Women Resident in Rural Areas of Northern Ghana.” Journal of Nutrition and Metabolism 2017: 1–10. 10.1155/2017/8497892. PMC5267082 28168052

[fsn371293-bib-0031] Sabina Azhar, B. , M. S. Islam , and M. R. Karim . 2021. “Prevalence of Anaemia and Associated Risk Factors Among Pregnant Women Attending Antenatal Care in Bangladesh: A Cross‐Sectional Study.” Primary Health Care Research & Development 22: e61. 10.1017/S146342362100061X. 34727999 PMC8569827

[fsn371293-bib-0032] Sebghati, M. , and E. Chandraharan . 2017. “An Update on the Risk Factors for and Management of Obstetric Haemorrhage.” Women's Health 13, no. 2: 34–40. 10.1177/1745505717716860. PMC5557181 28681676

[fsn371293-bib-0033] Stephen, G. , M. Mgongo , T. Hussein Hashim , J. Katanga , B. Stray‐Pedersen , and S. E. Msuya . 2018. “Anaemia in Pregnancy: Prevalence, Risk Factors, and Adverse Perinatal Outcomes in Northern Tanzania.” Anemia 2018: 1846280. 10.1155/2018/1846280. 29854446 PMC5954959

[fsn371293-bib-0034] Stevens, G. A. , M. M. Finucane , L. M. De‐Regil , et al. 2013. “Global, Regional, and National Trends in Haemoglobin Concentration and Prevalence of Total and Severe Anaemia in Children and Pregnant and Non‐Pregnant Women for 1995‐2011: A Systematic Analysis of Population‐Representative Data.” Lancet Global Health 1, no. 1: e16–e25. 10.1016/S2214-109X(13)70001-9. 25103581 PMC4547326

[fsn371293-bib-0035] Tettegah, E. , T. Hormenu , and N. I. Ebu‐Enyan . 2023. “Risk Factors Associated With Anaemia Among Pregnant Women in the Adaklu District, Ghana.” Frontiers in Global Women's Health 4, no. February: 1–10. 10.3389/fgwh.2023.1140867. PMC10902161 38425653

[fsn371293-bib-0036] Turawa, E. , O. Awotiwon , M. A. Dhansay , et al. 2021. “Prevalence of Anaemia, Iron Deficiency, and Iron Deficiency Anaemia in Women of Reproductive Age and Children Under 5 Years of Age in South Africa (1997–2021): A Systematic Review.” International Journal of Environmental Research and Public Health 18, no. 23: 12799. 10.3390/IJERPH182312799. 34886524 PMC8656986

[fsn371293-bib-0037] UNFPA . 2020. “Maternal Health.” 10.1007/978-1-4614-7918-5_6.

[fsn371293-bib-0038] UNICEF . 2021. “Maternal Mortality Rates and Statistics—UNICEF DATA.” Accessed March 31, 2025. UNICEF Data website. https://data.unicef.org/topic/maternal‐health/maternal‐mortality/.

[fsn371293-bib-0039] USAID . 2018. “Prevention of Maternal Anaemia.” Accessed August 3, 2022. https://www.mchip.net/interventions/maternal‐health/prevention‐maternal‐anemia/.

[fsn371293-bib-0040] Wemakor, A. 2019. “Prevalence and Determinants of Anaemia in Pregnant Women Receiving Antenatal Care at a Tertiary Referral Hospital in Northern Ghana.” BMC Pregnancy and Childbirth 19, no. 1: 495. 10.1186/s12884-019-2644-5. 31829146 PMC6907326

[fsn371293-bib-0043] WHO . 2014. “Anaemia—Definition.” Accessed December 5, 2024. https://www.who.int/health‐topics/anaemia#tab=tab_1.

[fsn371293-bib-0045] WHO . 2020. “Maternal Mortality.” Accessed August 2, 2022. https://www.who.int/news‐room/fact‐sheets/detail/maternal‐mortality.

[fsn371293-bib-0042] WHO . 2021. “Anaemia in Women and Children.” Accessed August 21, 2022. https://www.who.int/data/gho/data/themes/topics/anaemia_in_women_and_children.

[fsn371293-bib-0041] WHO . 2023. “Anaemia data.pdf.”

[fsn371293-bib-0044] WHO . 2024. “Guideline on Haemoglobin Cutoffs to Define Anaemia in Individuals and Populations.” In Sustainability (Switzerland) (11). WHO. https://www.ncbi.nlm.nih.gov/books/NBK602198/pdf/Bookshelf_NBK602198.pdf. 38530913

[fsn371293-bib-0047] World Medical Association . 1974. “WMA Declaration of Helsinki: Ethical Principles for Medical Research Involving Human Subjects.” 353, no. 1: 1418–1419. http://www.wma.net/en/30publications/10policies/b3/index.html.

