# Supplementary material for: Prevalence and Risk Factors of Iron Deficiency Anemia in Pregnancy in Ghana
Source: Food Sci Nutr. 2025 Nov 25;13(12):e71293. doi: 10.1002/fsn3.71293 (PMC12645153; doi:10.1002/fsn3.71293)
Supplement: Supplementary file 1 — Data S1: Supporting Information. [file FSN3-13-e71293-s001.docx]

**Table of contents**

**Title**

Prevalence and risk factors of iron deficiency anaemia in pregnancy in Ghana.

**Authors**

1. Betty Osei-Ntiamoah
2. Yvonne Nartey*
3. Kwadwo Ameyaw Korsah

**Key findings**

Iron deficiency anaemia in pregnancy remains highly prevalent and a threat to maternal health. Established risk factors are known worldwide; however, little is known about the risk factors specific to communities, hence the knowledge gap. The prevalence and risk factors of IDA in pregnancy were investigated in this study, with 81% as the overall prevalence among 394 participants. Unemployment, not taking malaria prophylaxis and having abnormal vaginal discharge were all associated with an increased risk of IDA.
